# Pigmented Eccrine Poroma in abdominal region, a rare presentation

**Published:** 2013-06-30

**Authors:** Monica Lorena Cárdenas, Claudia Juliana Díaz, Ricardo Rueda

**Affiliations:** a School of Dermatology and Dermatological Surgery, Department of Internal Medicine, Faculty of Health, Universidad del Valle, Cali, Colombia. E-mail: monilorena@hotmail.com; b Hospital Universitario del Valle, Department of Clinical Pathology, Cali, Colombia. E-mail: pcutanea@yahoo.com

**Keywords:** Poroma, abdomen, sweat gland neoplasms

## Abstract

The eccrine poroma or Hidracanthoma Simplex is a rare benign adnexal tumor of ephitelial cells, with an incidence of 0.001 to 0.008%1. In two-thirds of the patients it appears on the soles and lateral borders of the feet. We report the case of a patient with pigmented eccrine poroma in abdominal skin, of a rare entity presentation with a single report in the literature in that location.

## Abstract (es)

El poroma ecrino o hidroacantoma simple, es una tumoración anexial benigna de células epiteliales, de rara presentación, con una incidencia de 0.001 a 0.008%1. En dos tercios partes de los pacientes se presenta en la planta y bordes laterales de los pies. Reportamos el caso de una paciente con Poroma ecrino pigmentado en piel abdominal, entidad de rara presentación con un sólo reporte en la literatura en esa ubicación.

## Clinical case

**Figure 1:**
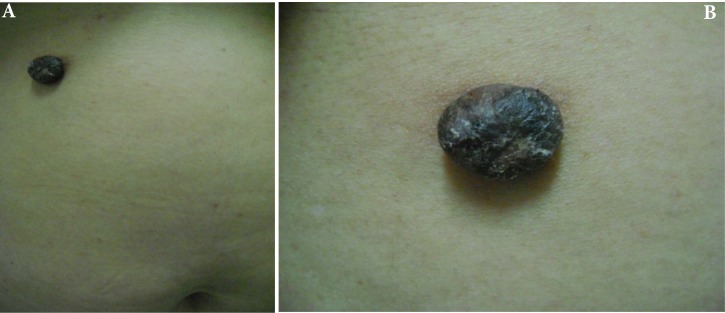
A and B. Hyperpigmented, hyperkeratotic nodule on abdominal skin. lesion Appear to be 0.5 mm long x 0.3mm wide in size

**Figure 2.**
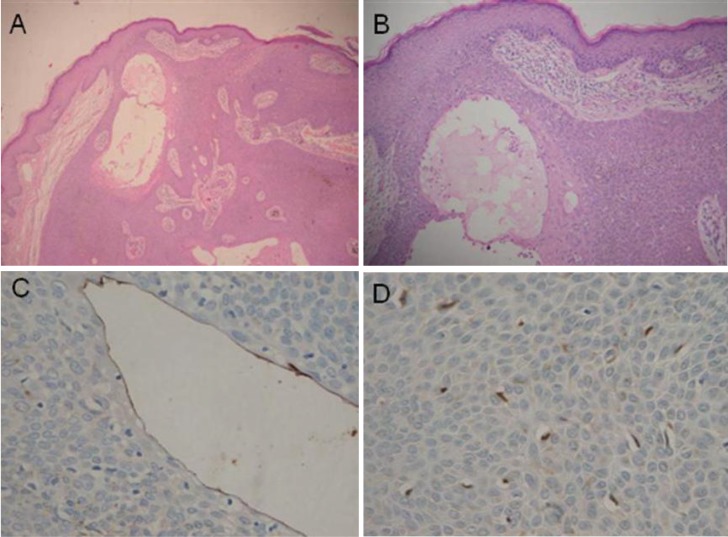
Hematoxylin and eosin staining, 10X and 40 x; Instrument Olimpus. Panel A and B are 10 x amplification, C and D are 40 x amplification. note proliferation of small keratinocytes emerging from the epithelium with epithelial tracts that anastomose within the dermis, some cavities lined by a eosinophilic cuticle (A,B), Carcinoembryonic Antigen (CEA) highlights positive in the elongated cells lining the cavities (C), the S100 marker is observed in dendritic cells and keratinocytes in the thickness of the cell proliferation (D).

Herein, we present a case of a 56-year-old female with a pigmented and painful nodule with progressive growth in the abdominal region with 15-year evolution, which presented easy bleeding in the last months prior to consultation. Upon physical exam, a hyperpigmented and angiomatous pedunculated nodule was found with hyperkeratotic surface (Fig 1). The following were considered as presumptive diagnoses: irritated seborrheic keratosis, epithelialized pyogenic granuloma, inflamed intradermal nevus, and nodular melanoma. A skin excision biopsy was carried out, whose report was a dermal tumor mass from the lower portion of the epidermis, well defined, formed by small cuboidal cells without atypia, arranged in well-defined bands that anastomose without barricade, with pigmented areas and few dilated ducts, which are in contact with the resection margins, compatible with eccrine poroma (Fig 2.) Immunohistochemistry was also performed, highlighting Carcinoembryonic Antigen (CEA) with elongated cells that line the cavities that support glandular differentiation. S100 staining was observed in the dendritic cells and melanocytes in the thickness of the cell proliferation (Fig 2).

The patient attended clinical controls post intervention, without lesion recurrence found until now.

## Discussion

Eccrine poroma is a benign adnexal tumor, of rare occurrence, with an incidence of 0.001 to 0.008%, first reported by Pinkus in 1956^1^. It originates as of the epithelium of the intra-epidermal portion of the eccrine duct. It typically appears as a papule or solitary nodule, euchromatic or red in the soles or lateral borders of the feet, corresponding to two-thirds of the cases; as with other eccrine gland tumors, a pigmented variant of rare presentation exists^2^ ^,^ ^3^; other sites affected are: the distal extremities, palms, and fingers, and less likely in the forearms, eyelids, thorax, scalp, external auditory canal, hip, gluteus, and abdomen^4^ ^-^ ^9^.

Melanocytes and melanin are rare in eccrine poroma and two hypotheses are posed regarding its presence originating the pigmentation: the first is that it comes from melanocytes present in cell primordia of eccrine ducts during the 14th week of gestation that were not eliminated during the maturity process; the other hypothesis is that it comes from epidermal melanocytes^10^.

This benign tumor has a favorable prognosis, without recurrence after complete resection and low risk of malignancy^1^.

## Conclusion

A case is presented of a patient diagnosed with pigmented eccrine poroma on abdominal skin, of unusual presentation and location, with only one case reported in the literature^10^.
